# Griffe cubitale d'origine lépreuse traitée par transfert tendineux de Lasso Zancolli: à propos d'un cas

**DOI:** 10.11604/pamj.2015.22.359.8374

**Published:** 2015-12-11

**Authors:** Adil El Alaoui, Mouhcine Sbiyaa, Aliou Bah, Ilyas Rabhi, Amine mezzani, Amine Marzouki, Fawzi Boutayeb

**Affiliations:** 1 Service de Chirurgie Orthopédique du Centre Hospitalier de Chambéry, France; 2 Service de Chirurgie Traumato-Orthopédie A, CHU Hassan II, Fès, Maroc

**Keywords:** Lèpre, griffe cubitale, transfert tendineux, Leprosis, ulnar claw, tendon transfer

## Abstract

La lèpre est une maladie infectieuse due à une mycobactérie (M. Leprae, Bacille de Hansen, ou BH) dont le tropisme nerveux est destructeur pour les cellules de Schwann. La localisation préférentielle des neuropathies tronculaire secondaire à la lèpre restent dominé par les zones ou les troncs nerveux traversent les défilés ostéo-ligamentaires inextensibles comme le défilé rétro-épitrochléen ou passe le nerf ulnaire. De nombreux travaux ont été consacrés à la souffrance nerveuse secondaire à la lèpre et surtout l'atteinte du nerf ulnaire qui se manifeste par une griffe des doigts. Le traitement dans ce cas est palliatif et fait appel à plusieurs techniques décrites dans la littérature. Nous rapportons dans ce travail un cas de griffe cubitale chez un patient lépreux traité par transfert tendineux de Lasso Zancolli.

## Introduction

Les paralysies des muscles intrinsèques des doigts secondaires à une atteinte lépreuse entrainent une déformation des doigts et une incapacité fonctionnelle de l'appareil musculaire des doigts. Cette déformation est une griffe des doigts longs qui se manifeste lors de l'extension active des doigts avec une hyperextension de la première phalange par déficit de stabilisation de la métacarpophalangienne alors que les deuxièmes et troisièmes phalanges restent en flexion, cette attitude ne concerne que le quatrième et cinquième doigt en cas de paralysie du nerf ulnaire isolée [1]. Nous allons rapporter l'observation d'un patient lépreux présentant une griffe cubitale, traitée chirurgicalement par transfert tendineux de Lasso Zancolli.

## Patient et observation

**Figure 1:**
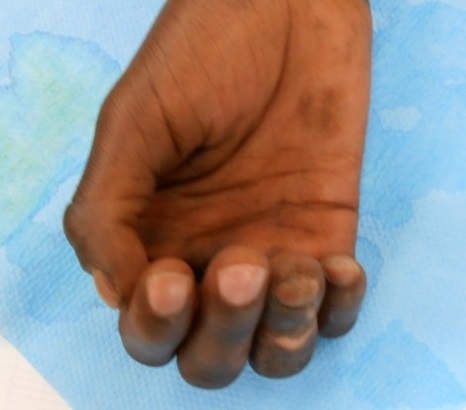
La main du patient avec les doigts en griffe cubitale

**Figure 2:**
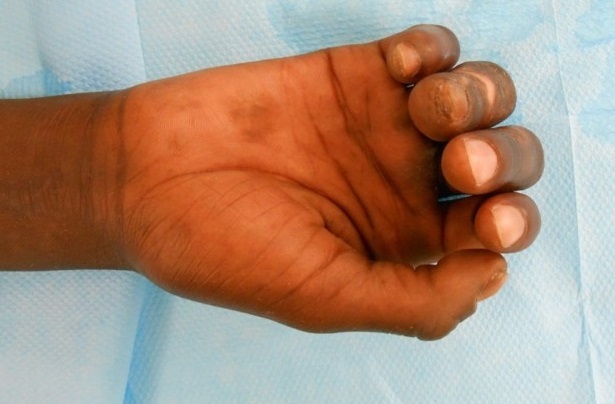
La main du patient après les séances de rééducation avec assouplissement du 3éme doigt

**Figure 3:**
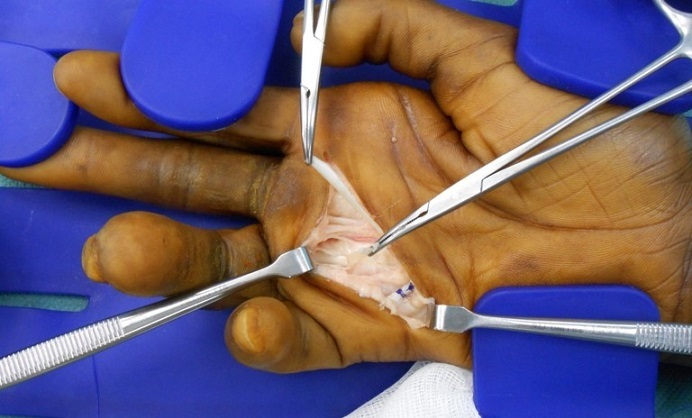
Image peropératoire montrant le premier temps du transfert tendineux avec section du fléchisseur commun superficiel des doigts

**Figure 4:**
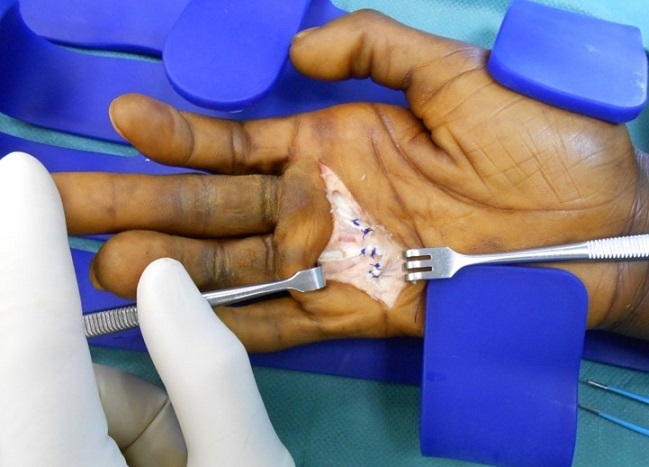
Image peropératoire montrant le 2éme temps opératoire après les sutures tendineuses en boucles autour de la poulie A1

Il s'agit d'un patient âgé de 33 ans droitier d'origine malienne, hospitalisé au service d'orthopédie pour prise en charge d'une griffe cubitale séquelle d'une atteinte lépreuse traité il y 6 ans. L'examen clinique trouve un aspect en griffe du 4éme et 5éme doigts de la main droite et des téguments très indurés en regard (Figure 1) et un signe de wartenberg positif, une raideur statique du 3éme, 4éme et 5éme doigts. L'examen vasculaire du poignet et de la main droite est sans particularités. L'examen électromyogramme trouve une paralysie sensitivomotrice du nerf ulnaire. Des séances de rééducation préopératoires ont été préconisé en premier temps pendant une durée de 6 semaines ce qui a donné de bon résultats avec récupération partielle de la souplesse du 3éme doigt, et assouplissement des téguments, alors que le 4éme et le 5éme doigts restaient raides (Figure 2). Nous avons pris chirurgicalement le patient pour corriger la déformation du 4éme et 5éme doigts. Sous anesthésie locorégionale du membre supérieure droit, par une incision transversale dans la paume de la main, on a procédé dans un premier temps à la section du fléchisseur commun superficiel (Figure 3) dans le canal digital en amant du chiasma de camper afin de respecter les vinculums qui assurent la vascularisation des fléchisseurs communs profonds des doigts et pour éviter un hématome source d'adhérences. Dans un deuxième temps on a réalisé une suture solide du fléchisseur commun superficielle à lui-même après un trajet en boucle autour de la poulie A1 (Figure 4) en laissant la métacarpo-phalangienne fléchie à 30°. Le patient a bénéficié d'une attelle plâtrée intrinsèque plus, bloquant que le poignet et les articulations métacarpophalangiennes pendant un mois. Une rééducation postopératoire a été réalisée pendant deux mois et a fait appel au début à des massages cutanés de la cicatrice avec utilisation des ultrasons. Après la sixième semaine, la sollicitation de l'appareil extenseur des quatrièmes et cinquièmes doigts s'est faite dans un premier temps sans résistance, puis en étirement (d'abord en flexion isolée de la métacarpo-phalangienne puis en flexion globale des doigts et du poignet). Lextension active métacarpo-phalangienne du quatrième et du cinquième doigt s'est faite en adduction avec des contraintes croissantes statiques au début puis dynamiques. À un an postopératoire, le résultat fonctionnel était bon avec absence de récidive du signe de Wartenberg et une extension active normale.

## Discussion

La griffe cubitale secondaire à une atteinte lépreuse observée lors de l'extension, est due à un déséquilibre entre les muscles intrinsèques non fonctionnels (paralysie ulnaire) et les extenseurs du quatrième et cinquième doigt selon Wartenberg [2], la correction de la griffe et la restauration de la flexion active métacarpo-phalangienne des doigts sont obtenues avec l'intervention choisie. Le premier objectif de la chirurgie est esthétique en supprimant la déformation en griffe. Le second objectif est fonctionnel en restaurant la flexion en volet des doigts longs, permettant la restauration de l'enroulement harmonieux des chaînes digitales. Une tension trop faible ne corrige pas correctement la griffe. Une tension trop forte peut induire des déformations des doigts en col-de cygne comme l'avait rapporté Littler [3, 4], Ce phénomène d'hypercorrection peut également être favorisé par l'hyperlaxité des chaînes digitales. Les neuropathies d'origine lépreuse concernant l'atteinte fréquente du nerf médian au poignet et du nerf cubital au coude entraînent des séquelles paralytiques qui entrent dans ce cadre particulier. De nombreuses techniques chirurgicales ont été proposées et ont montré leur efficacité avec 78% de bons et très bons résultats en moyenne [5], Les séries décrites dans la littérature sont rarement homogènes et non comparables. Les résultats analysés ne différencient pas toujours l'aspect esthétique (correction de la déformation en griffe) de l'aspect fonctionnel (restauration de la flexion active métacarpo-phalangienne) [6]. Lorsque l’étude est comparative, les résultats obtenus pour la restauration de la flexion active métacarpophalangienne sont meilleurs que les résultats obtenus pour la correction de la déformation en griffe des doigts longs. Dans les résultats publiés la moyenne des bons et très bons résultats 67,4% pour la correction de la déformation en griffe et 79,4% pour la restauration de la flexion active métacarpophalangienne [7, 8]. Parmi les techniques chirurgicales utilisées, la technique du lasso de Zancolli qui semble avoir les résultats plus homogènes avec 82% de bons et très bons résultats [9, 10]. Nous pensons que la différence des résultats peut sexpliquer par la souplesse, et par conséquent l'ancienneté, des déformations en griffe. L’épreuve décrite par Bouvier permet d'individualiser les griffes souples des griffes raides ou enraidies associant sur le même doigt long une raideur articulaire, rétraction tendineuse des fléchisseurs, détente tendineuse des extenseurs et raccourcissement cutané palmaire. Lorsque l’épreuve de Bouvier est négative (extension active incomplète ou impossible des interphalangiennes alors que l'articulation métacarpophalangienne est maintenue en flexion palmaire par l'examinateur) la griffe est dite raide [11, 12]. Si la griffe raide est partiellement réductible, la kinésithérapie permettra d'assouplir la raideur articulaire et de détendre les rétractions tendineuses et cutanées. Dans ce cas,la technique chirurgicale choisie devra plutôt s'orienter vers une restauration directe de la fonction des muscles intrinsèques avec le transfert d'un tendon sur la dossière des interosseux. Le choix du procédé chirurgical décrit par Zancolli nous semble un bon compromis. Dans le cas d'une griffe raide irréductible, un transfert nous semble dépassé. Il est alors licite de s'orienter vers une solution chirurgicale non conservatrice comme l'arthrodèse des articulations inter phalangiennes proximales.

## Conclusion

La chirurgie des griffes cubitales des doigts séquellaires d'une atteinte lépreuse est en la large diffusion surtout dans des pays à forte endémie de la lèpre. Son indication repose sur une analyse sémiologique précise de l'handicap ainsi que des besoins du patient. Sa réalisation suppose un environnement médico-chirurgical permettant une prise en charge du patient dans les phases pré et post-chirurgicales pour des soins de rééducation et d'appareillage qui font partie intégrante du traitement et qui conditionnent en grande partie la qualité du résultat final.
